# A novel GPR40 agonist, CNX-011-67, suppresses glucagon secretion in pancreatic islets under chronic glucolipotoxic conditions in vitro

**DOI:** 10.1186/1756-0500-7-595

**Published:** 2014-09-03

**Authors:** Mahesh Kumar Verma, Sanghamitra Biswas, Bhawna Chandravanshi, Korrapati Neelima, Anup M Oommen, Madanahalli R Jagannath, Baggavalli P Somesh

**Affiliations:** Connexios Life Sciences Private Ltd, No. 49, First Main road, 3rd phase, JP Nagar, Bangalore, 560 078 India

**Keywords:** GPR40, CNX-011-67, Glucagon, Glucolipotoxcity, GCG

## Abstract

**Background:**

Elevated glucose concentrations lead to increased insulin secretion and suppression of glucagon secretion. In fact, insulin is a physiological inhibitor of glucagon secretion. Type 2 diabetes mellitus (T2DM) patients have defects in insulin secretion. In addition to this, lack of suppression of glucagon secretion under elevated glucose concentrations is also observed in T2DM patients. We have earlier shown that GPR40 activation by CNX-011-67 stimulates glucose stimulated insulin secretion (GSIS). Here we extended our studies to examine the impact of GPR40 activation by CNX-011-67 on glucagon secretion from intact islets under both normal and glucolipotoxic conditions.

**Findings:**

Glucagon secretion from intact rat islets was suppressed under elevated glucose concentration. Activation of GPR40 by CNX-011-67 further suppressed glucagon secretion. Culturing islets under chronic glucolipotoxic (GL) conditions, we have observed increased high glucose mediated glucagon secretion and content which were reduced with GPR40 activation by CNX-011-67. Interestingly, expression of pre-proglucagon gene (GCG) remained unchanged under glucolipotoxicity in the presence or absence of GPR40 activation.

**Conclusion:**

Activation of GPR40 by CNX-011-67 can reduce glucagon secretion from pancreatic islets.

## Findings

Insulin and glucagon have opposing effects on blood glucose level and hence a rigorous control on their secretion is necessary for maintaining normoglycemia during both fasting and post-prandial states. In T2DM patients, impaired beta cell insulin secretion and impaired suppression of glucagon secretion from alpha cells lead to hyperglucagonemia mediated hyperglycemia [1, 2]. We have reported previously the development of CNX-011-67, a novel GPR40 agonist, which showed significant impact on beta cell health and insulin secretion [3]. Interestingly, Wang *et al.* [4] reported that GPR40 activation by free fatty acids could increase glucagon secretion in isolated pancreatic alpha-cells [4]. However, we did not observe any change in blood glucose levels in normal Wistar rats under fasting conditions by CNX-011-67 administration (unpublished data). Given that GPR40 can increase insulin secretion which is a physiological inhibitor of glucagon secretion; we presumed that its impact on intact islets could be different from what seen in isolated alpha cells. Since elevated glucose and insulin are known to suppress glucagon secretion [5], we hypothesized that activation of GPR40 by CNX-011-67 might also inhibit glucagon secretion in intact islets. To prove our hypothesis, we studied the impact of CNX-011-67 treatment on glucagon secretion in intact islets under both normal and glucolipotoxic condition.

### Acute treatment of CNX-011-67 reduces glucagon secretion

**Figure 1:**
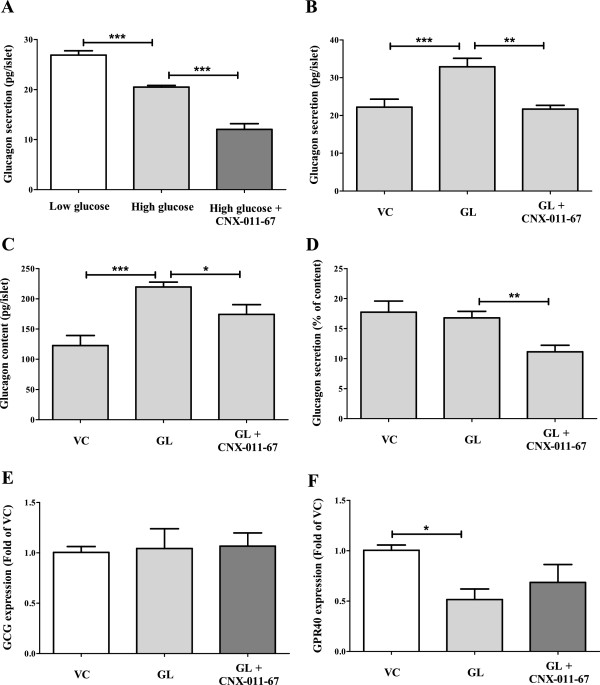
**Activation of GPR40 by CNX-011-67 treatment reduces glucagon secretion. (A)** Glucagon secretion was reduced under high glucose condition and was further reduced by CNX-011-67 treatment. After culturing islets under vehicle control (VC) or chronic glucolipotoxic (GL) conditions in presence or absence of CNX-011-67, islets were treated with high glucose concentration for 2 h and amount of secreted glucagon **(B)**, islet glucagon content **(C)** and glucagon secretion as % of content **(D)** were determined. After chronic culture, islets were used for gene expression analysis and mRNA levels of glucagon (GCG) **(E)** and GPR40 **(F)** were quantified. Data are represented as mean ± SEM from four replicates and statistical analyses were performed by ANOVA with Newman-Keuls post test (*P < 0.05, **P < 0.01 and ***P < 0.001).

Isolated rat islets exposed to high glucose concentration (11 mM) showed reduced glucagon secretion when compared to islets treated with low glucose (0.5 mM) (20.5 ± 0.3 *vs.* 26.9 ± 0.9 pg/islet, P < 0.001; Figure 1A). In presence of high glucose, CNX-011-67 treatment further reduced glucagon secretion (12.0 ± 1.1 *vs.* 20.5 ± 0.3 pg/islet, P < 0.001; Figure 1A). We earlier reported that CNX-011-67 treated islets showed enhanced insulin secretion in response to increasing glucose concentration [6]. Hence it is plausible that the enhanced insulin secretion by CNX-011-67 might have reduced glucagon secretion in isolated rat islets.

### Impact of CNX-011-67 treatment on glucagon secretion under chronic glucolipotoxic conditions

Intact rat islets were cultured under chronic glucolipotoxic (GL) conditions for 72 h to mimic T2DM pathology [7] followed by an acute exposure (2 h) to high glucose concentration. Using the same assay conditions, we have earlier shown that acute (2 h) high glucose induced insulin secretion was reduced under GL conditions and was restored by CNX-011-67 [3]. Exposure of islets to 11 mM glucose post 72 h treatment of GL condition elicited a higher glucagon secretion than that observed in control islets (32.9 ± 2.3 *vs.* 22.2 ± 2.1 pg/islet, P < 0.001, Figure 1B). However, chronic treatment with CNX-011-67 under GL conditions significantly reduced glucagon secretion (21.7 ± 1 *vs.* 32.9 ± 2.3 pg/islet under GL, P < 0.01; Figure 1B). Hence, CNX-011-67 reduced glucagon secretion under chronic GL conditions.

### Impact of CNX-011-67 treatment on islet glucagon content

An increase in glucagon content was observed in islets cultured under GL conditions (219.6 ± 8.1 *vs.* 122.7 ± 16.7 pg/islet under VC, P < 0.001; Figure 1C). Chronic activation of GPR40 by CNX-011-67 treatment under GL conditions resulted in a partial but significant reduction in glucagon content (174.4 ± 16.1 *vs.* 219.6 ± 8.1 pg/islet under GL, P < 0.05; Figure 1C). We earlier reported that under similar culture conditions CNX-011-67 increased insulin content [3].

When glucagon secretion was normalized to the islet glucagon content, we observed a significant reduction in glucagon secretion as % of glucagon content, only upon treatment with CNX-011-67 (11.1 ± 1.1 *vs.* 16.8 ± 1.1% of content, P < 0.01; Figure 1D).

### Glucolipotoxic conditions or CNX-011-67 treatment showed no impact on pre-proglucagon gene expression

Since glucagon content and secretion were reduced by CNX-011-67 treatment, we examined the impact on pre-proglucagon (GCG) gene expression. We did not observe any change in the expression of GCG in rat islets under GL conditions or by CNX-011-67 treatment (Figure 1E). Similar data were obtained from alpha-TC1, a pancreatic alpha-cell line (data not shown). In contrast to this unchanged GCG gene transcription by CNX-011-67 treatment, we previously reported that CNX-011-67 treatment reversed the reduction in insulin gene transcription caused by GL conditions [3].

### GPR40 gene expression under GL conditions or CNX-011-67 treatment

Islets cultured under GL conditions showed reduced GPR40/FFAR1 expression (0.51 ± 0.11 fold of VC, P < 0.05, Figure 1F). Treatment with CNX-011-67 could marginally increased its expression (0.69 ± 0.18 *vs.* 0.51 ± 0.11 fold under GL, P > 0.05, Figure 1F). These data show that CNX-011-67, which acts via activation of GPR40, has no significant impact on its expression.

Taken together, chronic GPR40 activation by CNX-011-67 treatment can reduce glucagon secretion and content without any change in GCG expression. However, a previous study with a GPR40 agonist failed to demonstrate any reduction in glucagon secretion. In that study even GLP1, a known inhibitor of glucagon secretion, failed to reduce glucagon secretion [8]. In order to find out whether GPR40 mediated impacts were primarily due to its action in alpha or beta cells, we measured its expression in both cell types by comparative gene expression analysis. We observed that GPR40 expression in an alpha cell line (alpha-TC1) was only 1.7% of that observed in a beta cell line (NIT1) (data not shown). These data are consistent with earlier finding [9] showing almost exclusive expression of GPR40 in beta cells and no expression in alpha cells. Interestingly, a similar pattern for GLP1 receptor expression in alpha and beta cells and its ability to increase insulin secretion and decrease glucagon secretion has been reported [10]. Moreover, during insulin granules exocytosis, ATP is also released from beta-cells which can inhibit glucagon secretion [11]. We have earlier shown that ATP levels are increased in islets after GPR40 activation by CNX-011-67 [6]. Hence suppressed glucagon secretion by GPR40 activation is mediated by the beta-cells secretory products but not by suppressing GCG expression. Hence, it appears that primary site of action for GPR40 is beta cells and its impacts on glucagon secretion are due to increased insulin granules exocytosis. Based on the very less expression of GPR40 in alpha cells and nature of control of insulin on glucagon secretion, we consider that CNX-011-67 mediated increase in insulin secretion is a possible mechanism to reduce glucagon secretion from intact islets. However, future studies are required for a definitive answer.

Taken together, our data provide evidences that GPR40 activation can suppress glucagon secretion under pathologically relevant condition thus providing potential benefit in reducing hyperglycemia.

## Methods

### Rat islet isolation, culture, treatment and gene expression analysis

Rat islets were isolated and were cultured either as control (vehicle control, VC) or under GL conditions (containing 16.7 mM glucose and 500 μM palmitate) for 72 h as described [7], in presence or absence of CNX-011-67 (0.3 μM). Gene expression studies were carried out as described [7]. Briefly, after 72 h of culture, islets were harvested in Tri-reagent (Invitrogen) and total RNA was isolated followed by first strand cDNA synthesis using reverse transcriptase and random hexamer (ABI, USA). Gene expression of target genes was measured using SYBR Green based PCR Master Mix (Eurogenetic, Belgium) and beta-actin was used as an internal control.

### Estimation of glucagons secretion and content

For estimation of glucagon secretion after acute exposure of CNX-011-67, rat islets were pretreated under low glucose condition (0.5 mM) in presence or absence of CNX-011-67 (0.1 μM) for 1 h. These islets were then incubated under low (0.5 mM) or high glucose (11 mM) for 2 h again in presence or absence of CNX-011-67. Amount of glucagon secreted in supernatant was then measured using glucagon ELISA kit (RnD Systems) as per manufacturer’s protocol.

For estimation of secreted and cellular glucagon after chronic treatment, rat islets were cultured under VC or GL conditions with or without 0.3 μM CNX-011-67 for 72 h. Islets were washed and pretreated under low glucose condition followed by incubation under high glucose for 2 h. The culture conditions were kept similar to our earlier study where we measured insulin secretion [3]. The supernatant was collected for estimating secreted glucagon and the islets were lysed for estimating cellular glucagon using ELISA kit (RnD Systems) and were represented as pg/islet.

### Statistical analyses

Data from four biological replicates are presented as mean with standard error of mean. For statistical analyses, ANOVA with Newman-Keuls post test was performed and P-values were calculated using GraphPad Prism. Statistical significance is represented as *P < 0.05, **P < 0.01 and ***P < 0.001.

## Abbreviations

GPR40: G protein coupled receptor 40
T2DM: Type 2 diabetes mellitus
VC: Vehicle control
GL: Glucolipotoxicity
GLP1: Glucagon like peptide 1
GCG: Glucagon gene.
